# Gene Specific Actions of Thyroid Hormone Receptor Subtypes

**DOI:** 10.1371/journal.pone.0052407

**Published:** 2013-01-03

**Authors:** Jean Z. Lin, Douglas H. Sieglaff, Chaoshen Yuan, Jing Su, AnithaChristy S. Arumanayagam, Sharareh Firouzbakht, Jaime J. Cantu Pompa, Frances Denoto Reynolds, Xiabo Zhou, Aleksandra Cvoro, Paul Webb

**Affiliations:** 1 Genomic Medicine, The Methodist Hospital Research Institute, Houston, Texas, United States of America; 2 Department of Biology and Biochemistry, Center for Nuclear Receptors and Cell Signaling, University of Houston, Houston, Texas, United States of America; 3 Diabetes Center, University of California Medical Center, San Francisco, California, United States of America; 4 Radiology Department, The Methodist Hospital Research Institute, Houston, Texas, United States of America; 5 Department of Research and Innovation, Tec de Monterrey School of Medicine and Health Science, Monterrey, Mexico; Beckman Research Institute of City of Hope, United States of America

## Abstract

There are two homologous thyroid hormone (TH) receptors (TRs α and β), which are members of the nuclear hormone receptor (NR) family. While TRs regulate different processes *in vivo* and other highly related NRs regulate distinct gene sets, initial studies of TR action revealed near complete overlaps in their actions at the level of individual genes. Here, we assessed the extent that TRα and TRβ differ in target gene regulation by comparing effects of equal levels of stably expressed exogenous TRs +/− T_3_ in two cell backgrounds (HepG2 and HeLa). We find that hundreds of genes respond to T_3_ or to unliganded TRs in both cell types, but were not able to detect verifiable examples of completely TR subtype-specific gene regulation. TR actions are, however, far from identical and we detect TR subtype-specific effects on global T_3_ response kinetics in HepG2 cells and many examples of TR subtype specificity at the level of individual genes, including effects on magnitude of response to TR +/− T_3_, TR regulation patterns and T_3_ dose response. Cycloheximide (CHX) treatment confirms that at least some differential effects involve verifiable direct TR target genes. TR subtype/gene-specific effects emerge in the context of widespread variation in target gene response and we suggest that gene-selective effects on mechanism of TR action highlight differences in TR subtype function that emerge in the environment of specific genes. We propose that differential TR actions could influence physiologic and pharmacologic responses to THs and selective TR modulators (STRMs).

## Introduction

Thyroid hormone (TH) receptors (TRs α and β) are highly homologous transcription factors which transduce signals of active forms of TH (predominantly tri-iodothyronine, T_3_) [1], [2]. Like other nuclear receptors (NRs), TRs bind specific DNA response elements (TREs) comprised of degenerate repeats of the sequence AGGTCA, usually as heterodimers with retinoid X receptors (RXRs). From these locations, the TRs recruit coregulator complexes that influence gene expression and T_3_ modulates transcription by inducing conformational changes in the receptor C-terminal ligand binding domain which, in turn, alters the complement of TR associated coregulators [3]–[5]. Despite similarities in structure and function, analysis of TR gene knockout mice and human patients with TRα or TRβ mutations has revealed that the two TRs display unique activities *in vivo* [6]–[11]. TRα plays major roles in regulation of heart rate and muscle whereas TRβ modulates serum cholesterol levels via actions in liver and feedback inhibition of TH production through the hypothalamic-pituitary-thyroid axis. TRs also exhibit subtype-specific effects in regulation of basal metabolic rate, bone development and other processes.

Differential effects of TRs have commonly been attributed to variations in TR expression levels in target tissues. Liver, for example, expresses TRβ and TRα in a 5∶2 ratio and this could explain the predominant role of TRβ in cholesterol metabolism [12], [13]. Indeed, comparison of T_3_ effects in hypothyroid wild type, TRα, TRβ and double TR knockout mouse livers failed to identify truly TR subtype selective genes [14]. It is noteworthy, however, that other closely related NRs, including estrogen receptors (ERs) α and β, regulate quite different gene sets [15]. Given that current approaches to selectively modulate TH signaling pathways have often focused on development of TR subtype selective modulators (STRMs) [12], it is important to assess the extent of TR subtype-specific effects on gene regulation.

Several pieces of evidence suggest that TRα and TRβ actions may not be absolutely identical at the level of individual target genes. First, stably transfected TRα and TRβ regulate the same set of around 40 target genes in HepG2 liver cells, but with significant subtype specificity in magnitude of T_3_ response at individual genes [16]. Second, TRα and TRβ exhibit similar effects at transfected reporters with the most common form of TRE (direct repeat spaced by 4 nucleotides, DR-4), but TRβ is more active at inverted palindromic (IP) TREs [17]. Third, detailed qPCR analysis of expression of liver genes in TR knockout mice has revealed apparently exclusively TRβ-dependent T_3_ regulated genes, including *thrsp* and *angptl3* [18]. Fourth, TR subtypes exhibit differential effects on ABCD2 gene via indirect differential effects on expression of the transcription factor SREBP1c [19]. Fifth, a constitutively unliganded TRα mutant represses transcription of the liver C/EBPβ gene more efficiently than an equivalent TRβ mutant, via differential corepressor recruitment [20]. Finally, TRβ selectively represses the thyroid stimulating hormone (TSH) gene in cultured pituitary cells even though both TRs are present and TRα is functional when expression of TRβ is knocked down [21].

In addition to TR subtype specificity, there is evidence that activity of both TRs is highly gene-context dependent [1], [14]. T_3_ can either activate or repress transcription of target genes but, within this framework, there are differences in contributions of unliganded and liganded TRs. Commonly, unliganded TRs suppress T_3_-inducible genes and agonists induce transcription by both reversing the inhibitory effects of unliganded TRs and eliciting further transcriptional activation. There are, however, cases in which unliganded TRs fail to suppress T_3_ induced genes or where T_3_ activates genes solely by relieving unliganded TR-dependent basal repression [14], [16]. Similar gene-specific variations in the balance of unliganded and liganded TR actions also occur at negatively regulated genes and TRs can even exhibit completely ligand-independent actions [14], [16]. T_3_ concentration dependence also varies; euthyroid T_3_ levels are sufficient for optimal induction of some genes whereas others need higher (hyperthyroid) T_3_ levels [14], [22].

Here, we set out to define the extent of TR subtype and gene-specific variations in transcriptional response by creating cells with similar levels of TRα or TRβ. While our data suggests that there are no completely TR subtype specific genes, we observed TR-subtype and cell-specific effects on the kinetics and magnitude of transcriptional response, patterns of TR-dependent gene expression and T_3_ concentration dependence that verify and extend conclusions of previous groups. We discuss possible mechanisms of these differential effects and their impacts upon physiological responses to THs and actions of STRMs.

## Materials and Methods

### Reagents

Triiodothyronine (T_3_) suitable for cell culture was purchased from Sigma Aldrich (T6397).

### Construction of HepG2-TR Cells

HepG2 cells were grown in Dulbecco's modified Eagle's medium (DMEM) supplemented with 10% fetal bovine serum (FBS), 100 U/mL of penicillin, 0.1 g/L of streptomycin and 4 mmol/L glutamine, under 95% air and 5% CO_2_ at 37°C. Lentiviral vector pSicoR containing GFP was a gift from the McManus laboratory (UCSF, http://mcmanuslab.ucsf.edu/). Sequences for appropriate tags were added to TR cDNAs derived from previously described eukaryotic expression vectors by standard PCR amplification and resulting hybrid cDNAs were cloned into pSicoR vector at a location C-terminal of GFP. An oligonucleotide encoding a T2A peptide that will mediate co-translational cleavage of protein was inserted between GFP and TRs coding sequences to facilitate independent expression of GFP and TRs. pSicoR vectors containing tagged TRs were transfected into HEK 293 cells to create packaged virus particles by the Diabetes center core lab at UCSF. The virus was titrated by GFP fluorescence. HepG2 cells grown on a 6-well plate were transduced with lentivirus particles containing tagged TR sequences. After incubation with virus particles for 72 hours, cell culture media were replaced with regular growth media and GFP expression was verified by fluorescent microscopy. Cells were split upon confluence and sorted by fluorescence-activated cell sorting in UCSF core facility after two passages. Clones of GFP positive cells were collected and grown in 10 cm plates. When cells reached confluence, TR expression was analyzed with Western blots using anti-tag antibodies and transient transfection of TRE driven luciferase reporters, described below. Cells were maintained in regular growth medium as above.

### Construction of HeLa-TR cells

HeLa cells were grown in similar conditions to HepG2 cells. HeLa cells stably expressing tagged TRα and TRβ were generated using the Tet-Off gene expression system (Clontech). Stable clones were selected by hygromycin-resistance (400 µg/ml) and screened for TR protein expression. HeLa cells expressing TRβ and TRα were maintained in media supplemented with doxycycline (20 ng/ml).

### Western Analysis

HepG2 or HeLa cells were lysed using Triton X-100 lysis buffer. Cell extracts (10 µg of total protein) were separated by SDS-PAGE and transfer onto a PVDF membrane. Transfer membranes were then incubated with anti-flag M2 antibody (Sigma-Aldrich) or c-Myc antibody (Clontech) at a 1∶1000 dilution at 4°C for 16 h followed by goat anti-mouse IgG-horseradish peroxidase antibody (Santa Cruz Biotechnology sc-2004) at a 1∶10000 dilution for 45 min at room temperature. Blots were visualized by applying ECL Plus (GE Healthcare).

### Hormone Binding

Hormone binding assays were carried out as described in [23]. The *K_d_* values were calculated using the Graph-Pad Prism computer program (Graph-Pad Software Inc).

### Transfection

Cells were co-transfected with a DR-4 or IP-6 TRE-driven luciferase reporter and constitutive *renilla* luciferase reporter (Promega) using Transfectin Reagent (BioRad) and plated in 12-well plates in growth medium (DMEM with 10% hormone-depleted FBS) [23]. After 16 h of incubation, T_3_ (100 nM) or vehicle (DMSO) was added in triplicate. After an additional 24 h of incubation, cells were harvested and assayed for luciferase activity using the Promega Dual Luciferase Reporter Assay (Promega). Data were normalized to the *renilla* luciferase activity.

### mRNA and cDNA Preparation

For HepG2, total RNA was prepared using the Aurum Total RNA kit (Bio-Rad). Reverse transcription reactions in these samples were performed using 1 µg of total RNA with an iScript cDNA Synthesis kit (Bio-Rad). Total RNA concentrations were measured using NanoDrop ND-1000 spectrophometer. For HeLa, total RNA was extracted from cells with Qiazol Lysis Reagent (Invitrogen) and purified with RNeasy® Mini kit (Qiagen) following manufacturer's instructions. mRNA was reverse transcribed into cDNA with a mixture Oligo(dT)_20_ and Random Hexamers (1∶1 ratio) using SuperScript™ III First-Strand Synthesis System for RT-PCR kit (Invitrogen).

### Microarray hybridization

Human whole genome expression arrays were purchased from Illumina (Human WG-6v2 and Human WG-6v3). cRNA synthesis and labeling were performed using Illumina® TotalPrep™-96 RNA Amplification Kit (Ambion). Labeling *in vitro* transcription reaction was performed at 37°C for 14 h. Biotinylated cRNA samples were hybridized to arrays at 58°C for 18 h according to manufacturer's protocol. Arrays were scanned using BeadArray Reader.

### Statistical analysis

Unmodified microarray data obtained from GenomeStudio was background-subtracted and quantile-normalized using the lumi package [24] and analyzed with the limma package [25] within R [26]. To determine the effect of TRα and TRβ over-expression in the absence of ligand (“unliganded-effect”), cell lines were analyzed separately by LIMMA (“parental”, with no exogenous TRs, TRα, and TRβ), followed by contrast analysis. To better determine TR isoform effects, factorial LIMMA analysis was conducted comparing ligand (T_3_) with over-expression of the TRα or TRβ (“TR over-expression with ligand effect”; interaction between T_3_ and over-expression of TRα or TRβ), followed by contrast analysis. All analysis was corrected for multiple hypothesis testing [27], and effects determined to be significant when ≥2-fold with an adjusted p-value ≤ 0.05 when compared to their respective parental cell line. To facilitate comparisons among the various datasets, all data was uploaded into a SQLite3 database [28]. Heatmaps were produced and clustered using multiarray viewer [29].

### qPCR

Real-time qPCR in HepG2 samples was performed with the Roche LightCycler 480 RT PCR Instrument using SYBR Green Mastermix (Roche). The sequences of the primers are listed in Table S1. The data were collected and analyzed using the comparative threshold cycle method. Experiments were performed at least three times, and the mean ± SE was calculated using the Prism curve-fitting program (GraphPad Software, version 3.03; GraphPad). For HeLa, qPCR was performed using SYBR Green PCR Master Mix (Applied Biosystems) on ABI 7900HT RT-PCR system (Applied Biosystems) with default two-step QRT-PCR program. Amplification curves were evaluated by the comparative C_t_ analyses.

### Calculation for categorizations of expression patterns

Custom Python scripts were used to organize the expression patterns into induced and repressed effects, through the calculation of minimal Euclidean distances between nine hypothetical patterns (2-by-3; unliganded and liganded effect expressed as induced (e.g., 2-fold), repressed (e.g., 0.5-fold) or no effect (e.g., 1)) and that experimentally derived. The hypothetical patterns (unliganded, ligand) were: pattern RR = 0.5, 0.5; pattern RO = 0.5, 1; pattern RI = 0.5, 2; pattern OR = 1, 0.5; pattern OO = 1, 1; pattern OI = 1, 2; pattern IR = 2, 0.5; pattern IO; 2, 1; pattern II = 2, 2. Note, hypothetical pattern OO translates to no substantial effect with TR over-expression (unliganded effect) or T_3_-treatment (ligand effect) and was not included in the final table. All probes with a BH adjusted p-value< = 0.05 within the 3 hr HepG2 and 24 hr Hela treatments were analyzed. To lessen the effect of extremes, fold-change values were first transformed to 2 or 0.5 if their fold-change values were >2 or <0.5, respectively. Euclidean distances were then calculated, and the probe/gene transcript grouped into the hypothetical pattern that delivered the minimal Euclidean distance between the experimental and/or the transformed vector and the hypothetical pattern vector. The genes were subsequently translated from Probe_ID to official gene name using an SQLite database.

## Results

### Cells that Express Comparable Levels of TRα1 or TRβ1

**Figure 1:**
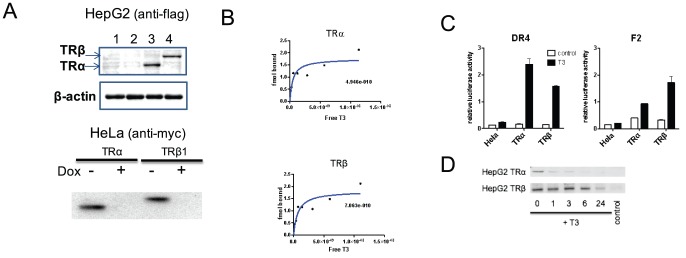
Cells that express TRs. **A**. Equal expression of exogenously expressed TRs. Upper panel, western blot of extracts of HepG2 parental cells (1), HepG2 cells infected with control lentivirus (2) or cells infected with lentivirus expressing TRα (3) or TRβ (4) and blotted with anti-flag antibody. Inset beneath shows the same extracts blotted with a β-actin antibody as a loading control. Lower panel; western blot of HeLa-TR extracts after +/− doxycycline withdrawal to induce TRs and blotted with anti-myc. **B**. Results of T_3_ binding assays performed on extracts of HeLa-TR cells after 24 hrs doxycycline withdrawal; figures in panels represent deduced affinities of expressed TRs for T_3_. **C**. Results of luciferase assays performed upon HeLa-TR cells transfected with standard TRE-driven reporters, DR-4 Luc and IP-6 (F2)-Luc after doxyclycline withdrawal to induce TR expression. **D**. Western blot of HepG2-TR extracts at various times after initial T_3_ treatment.

We created two sets of TR-expressing cells to compare actions of major TR subtypes at endogenous genes. For HepG2 liver cells, we used a retroviral infection to express epitope-tagged (Flag) TRα1 or TRβ1. For HeLa, we used stable transfection to express epitope-tagged (myc) TRs under tetracycline control (tet-off system, Fig. S1). We screened multiple clones of both cell types by western, using antibodies against respective epitope tags to facilitate direct comparisons of protein levels, and identified pairs of cell lines with comparable TRα and TRβ expression (Fig. 1A). We confirmed that exogenous TRs were recognized by antibodies against TR primary sequences, that these TRs were expressed at higher levels than endogenous TRs, which were either present at very low levels (TRβ in HepG2 [22]) or undetectable (TRα in HepG2 and both TRs in HeLa) and that expressed TRs were of correct molecular weight (Fig. S2 and not shown). We also verified that TR expression was stable over several passages (not shown).

Exogenously expressed TRs are functional. We verified that the presence of the tag did not affect TR function in transient transfections, in which we compared native and tagged TRs (Fig. S3 and not shown). We were also unable to detect any major differences between the function of exogenously expressed TRs versus previous assessments of endogenous TR function. Hormone binding analysis confirmed that TRs exhibit affinities for T_3_ that are consistent with previously reported values (Fig. 1B and not shown). This data also allowed us to estimate numbers of T_3_ binding sites per cell (i.e. TRs), which were between 7-10,000 in transfected cells versus essentially undetectable in parental cells [22] and within physiological range (<10,000 receptors per cell) [30]. Exogenous TRs conferred T_3_ responses on standard TRE-dependent reporters in both cell types (Fig. 1C and not shown). Further, T_3_ elicited similar levels of activation with TRα and TRβ at a DR-4 reporter but larger levels of activation with TRβ at an IP-6 reporter, in accordance with previous results [17]. Finally, TR steady state levels were diminished after T_3_ treatment (Fig. 1D); this phenomenon is common to many NRs and a consequence of ubiquitin-dependent turnover of activated receptors [31]. Interestingly, however, TRα levels were rapidly reduced (within 1 hour of T_3_ addition) whereas TRβ levels only obviously became diminished after extended ligand treatments.

### TRα and TRβ Regulate Similar Gene Sets in HepG2 with Different Kinetics

We performed transcriptome wide analysis of TR target genes in our HepG2-TR cells and parental HepG2 controls after 3, 6 and 24 hr induction with saturating (100 nM) T_3_. Since HepG2 expresses low levels of endogenous TRβ1, we examined interaction between treatment and cell line (i.e. T_3_ + TR over-expression) to determine TR-specific effects.

**Figure 2:**
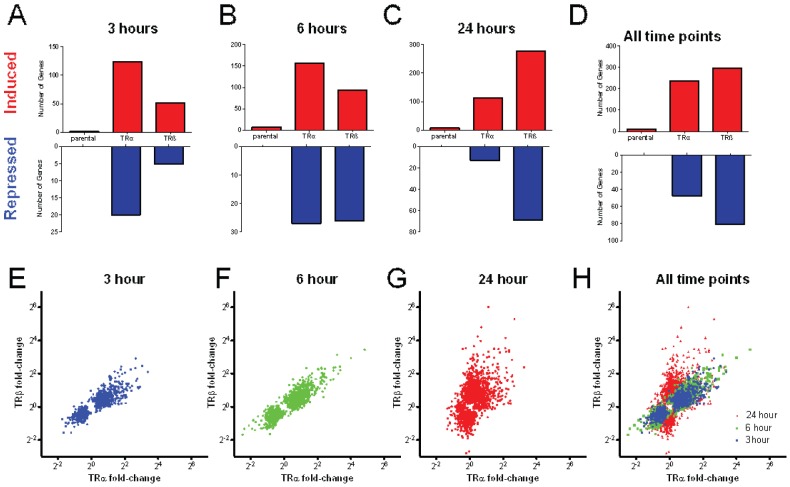
T_3_ Response in HepG2 cells. **A–D**. Numbers of genes that meet cut-offs for fold induction and statistical significance in parental HepG2, HepG2-TRα or HepG2-TRβ at each time point, **A**, 3 hr, **B**, 6 hr, **C**, 24 hr, **D**, all three time points combined. T_3_ induced genes are represented in upper panels (red) and T_3_ repressed genes in lower panels (blue), note the difference in scale which means that many more genes are positively regulated than negatively regulated. **E–H**. Plots of fold induction/repression by T_3_ in the presence of TRβ (y-axis) versus TRα (x-axis). **E**, 3 hr blue, **F**, 6 hr green, **G**, 24 hr red, **H**, all three time points.

**Figure 3:**
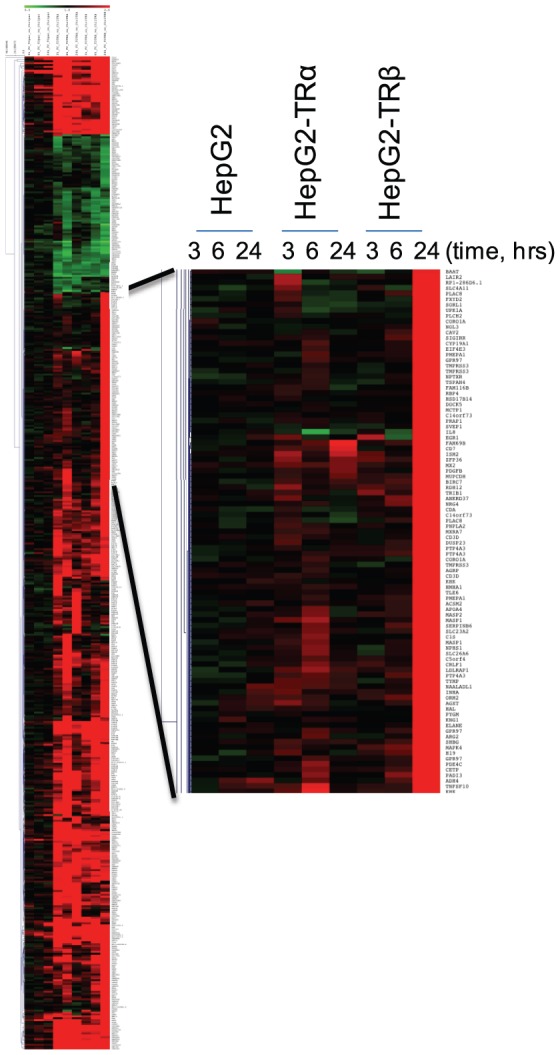
Heatmap to Illustrate Patterns of T_3_ Response in HepG2. **A**. Representation of changes in all T_3_-dependent genes that meet cutoffs for fold induction/statistical significance in HepG2 parental cells and HepG2-TRα or HepG2-TRβ cells at each time point. Red, upregulated, green, downregulated, black, no change. Genes were clustered according to similarities in response patterns as described in Methods. **B**. As for Fig. 3A, with a section of the heatmap expanded to reveal one of the clusters of late emerging TRβ-preferential T_3_ responses.

Most T_3_ responses required exogenous TRs (Fig. 2A–D, Fig. 3). As we previously reported [22], a few genes responded to T_3_ in parental HepG2 cells, with around 17 meeting cut-offs (>2.0 fold, BH-adjusted P value 0.05) at 24 hrs. This is due to vanishingly low levels of functional TRβ present in HepG2 [22]. By contrast, hundreds of genes responded to T_3_ in cells that express either of the two TRs (Fig. 2A–D). Of these, the majority (more than 70%) were induced by T_3_ with the remainder repressed. Additionally, most genes that exhibited T_3_ responses in parental HepG2 cells exhibited amplified responses in the presence of exogenous TRs. The sole exception was that we discovered expression of the highly T_3_ responsive ANGPTL4 gene, a verified direct TRβ target in parental HepG2 cells [22], was silenced by exogenous TRs when we performed qPCR analysis (Fig. S4). We confirmed that enhanced T_3_ responses seen in the presence of transfected TRs in HepG2 were dependent upon exogenous TR expression using an siRNA specific to the 5′ portion of the dual EGFP/TR transcript to inhibit exogenous TR expression (Fig. S5).

Unexpectedly, different numbers of genes met cutoffs for fold induction and statistical significance with TRα and TRβ at each of the three times (Fig. 2A–C). More T_3_ responsive genes appeared with TRα at 3 and 6 hrs (Fig. 2A, B), whereas TRβ responses predominated at 24 hrs (Fig. 2C). Overall, similar numbers of genes exhibited T_3_ -responses when all three times were considered together (Fig. 2D). Closer analysis revealed no completely TR subtype-specific genes within the datasets, there was a high degree of overlap between TRα and TRβ responsive genes and nearly all genes that responded to T_3_ with either TRα or TRβ at any of the three time points exhibited qualitatively similar responses with the other TR in at least one time point (Fig. 3A and not shown).

**Figure 4:**
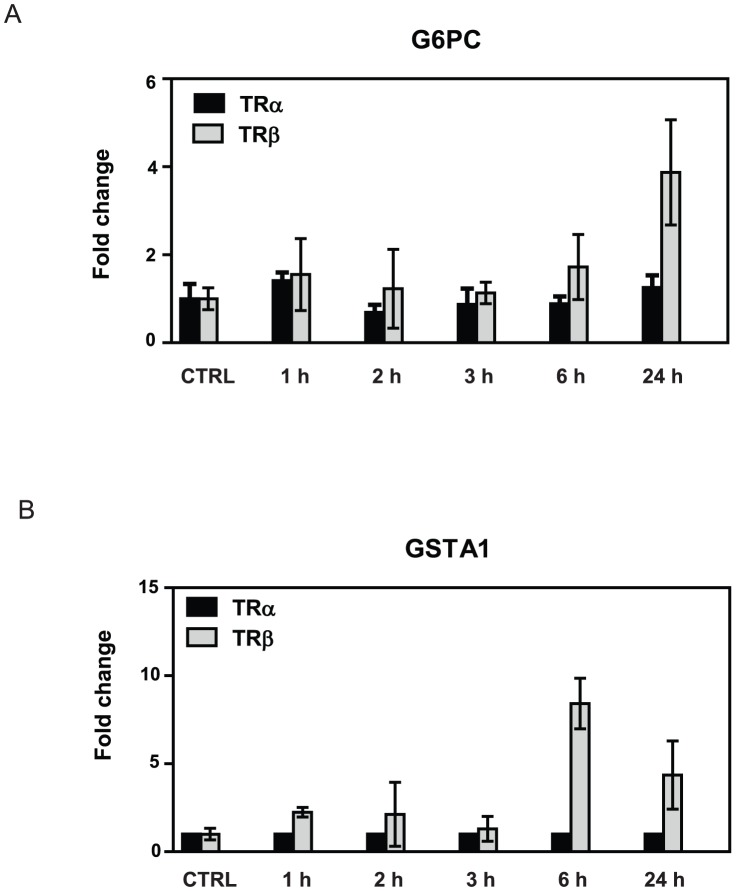
Verification of TRβ preference of late responding genes. qPCR analysis of T_3_ induction of two genes (**A**, G6Pc and **B**, GSTA1) identified as preferential late TRβ responders. Note that TRα-responses are weak and that TRβ preference persists across all timepoints.

To better understand differential kinetics of T_3_ response in HepG2-TRα1 cells and HepG2-TRβ1 cells, we compared fold T_3_ induction/repression of each gene (Probe_ID) in the presence of the two TRs (Fig. 2E–H). Although there were more TRα selective genes at 3 and 6 hrs, there was a strong apparent correlation between fold induction/repression when the two TRs are compared (Fig. 2E, F). Visual inspection (Fig. 3A, not shown) and statistical correlation analysis suggested that many of the apparently TRα-selective genes responded in a similar fashion to TRβ, but that T_3_ response sometimes failed to meet cutoffs for fold induction and/or statistical significance resulting in the discrepancies between numbers of regulated genes. While there was also apparent correlation between overall TRα and TRβ responses at 24 hrs (Figs. 2G, H; Fig. 3A, B), we observed a shift in slope that reflected an increase in the number of genes (probes) responding to T_3_ in the presence of TRβ versus TRα, resulting in a deviation from the straight line relationship at the earlier time points. The latter phenomenon reflected emergence of a subset of genes with preferential TRβ responses, although all members of this gene class exhibited qualitatively similar regulation by TRα in at least one earlier time point (see Fig. 3B). We used qRT-PCR analysis to confirm that members of this strongly TRβ-dependent late responding gene set (G6Pc, GSTA1) retained preferential TRβ responses that persisted over multiple T_3_ incubation times (Fig. 4).

Together, our data suggests that TRs regulate similar gene sets but with different kinetics in HepG2; T_3_ responses emerge earlier with TRα versus TRβ. Further, a strongly TRβ-dependent subset appears after prolonged T_3_ treatment.

### T_3_-induced Genes are Direct TR Targets

**Figure 5:**
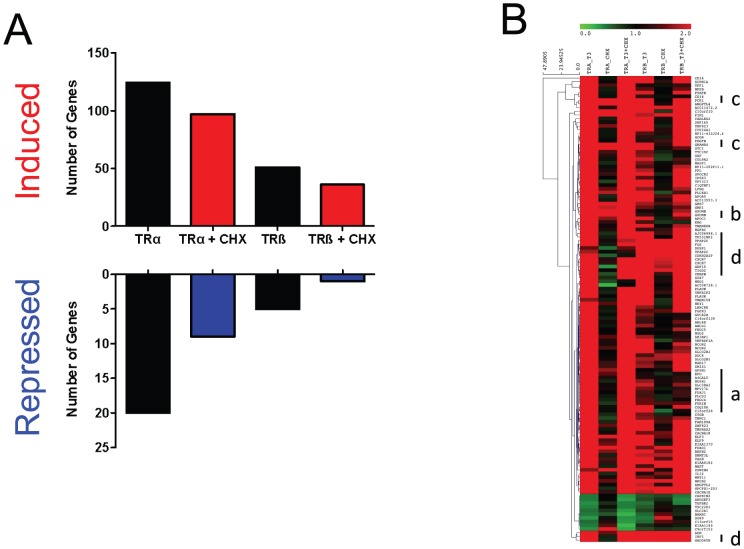
T_3_ Induced Genes are Direct TR Targets. **A**. Bar graph representing numbers of T_3_ induced (upper panels) and repressed (lower panels) genes at 3 hrs in HepG2-TRα and HepG2-TRβ cells that persist with CHX pre-treatment (upper panel, red, lower panel, blue). **B**. Heat map representing gene expression changes at 3 hrs timepoint in HepG2-TRα and HepG2-TRβ cells with T_3_, CHX and T_3_ + CHX. Note that most target genes retain their T_3_ responses with CHX. Examples of genes with unusual responses are marked by lower case letters: a = stronger T_3_ responses with TRα, b = amplification of weak T_3_ responses in the presence of TRβ with CHX, c = selective CHX-dependent gene induction in the presence of TRα, d = selective CHX-dependent gene induction in the presence of TRβ.

We determined the proportion of T_3_ responsive genes that were direct TR targets (Fig. 5). To do this, we examined effects of pretreatment with the protein synthesis inhibitor cycloheximide (CHX) upon T_3_ response at 3 h and defined direct targets by persistence (at least 100%) of gene expression obtained with T_3_ after CHX treatment relative to levels obtained with T_3_ alone. Most (>80%) of the genes that are positively regulated by T_3_ are scored as direct targets by these criteria (Fig. 5A upper panel). Investigation of remaining positively regulated genes revealed that some T_3_ activation persisted in the presence of CHX for many of the remaining 20% of genes, suggesting that representation of direct TR targets within this dataset may be even larger than this analysis suggests (Fig. 5B, not shown). Interestingly, a much smaller proportion of negative T_3_ responses persisted after CHX treatment versus positive responses (Fig. 5A, lower panel). This suggests that novel protein synthesis is needed for T_3_ repression in this cellular context. In total, CHX treatment only completely abolished T_3_ response of a small subset of genes (Fig. S6); responses of ≈11% of all genes that displayed 2- or greater fold responses to T_3_ at 3 hrs were completely inhibited by CHX. These are likely to represent secondary responses to T_3_-dependent changes in protein levels.

As seen with the complete dataset, we observed little TR subtype selectivity among direct TR targets (Fig. 5B). Some T_3_ responsive genes display stronger TRα responses and qualitatively similar but weaker responses with TRβ; this is evident from comparison of columns 1 and 4 in the heat map (some examples of this set of genes marked “a”). However, T_3_ responses mostly persisted with CHX in the presence of both TRs and we even detected cases of amplification of weak T_3_ response with TRβ in the presence of CHX (examples of this set of genes marked “b”). There were also gene-specific interactions of TRs and CHX; some genes were selectively de-repressed by CHX treatment alone in the presence of one of the two TRs (TRα selective de-repression is marked “c” and TRβ selective de-repression marked “d”). In general, however, most direct target genes appear similarly regulated by both TRs.

### TR/Gene-Selectivity in T_3_-response

**Figure 6:**
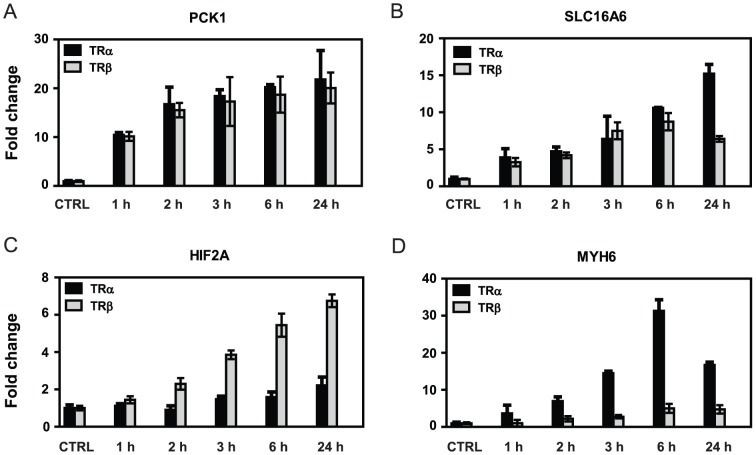
Verification of different T_3_ response patterns in HepG2 cells. Results of qPCR analysis of representative gene expression changes at various times after T_3_ induction in HepG2-TRα and HepG2-TRβ cells. **A**, PCK1, similar with both TRs, **B**, SLCA16A, similar with both TRs at most times, **C**, HIF2A, TRβ preference at both early and late times, **D**, Myh6, TRα preference at early and late times.

Within broad TR response patterns outlined above, there was gene-specific variability of T_3_ regulation patterns and we verified some of these observations using RT- qPCR (Fig. 6). Many genes displayed similar time courses of T_3_ induction with both TRs (PCK1, Fig. 6A), but others exhibited differential responses to the at individual time points (SLC16A6, Fig. 6B) and yet others displayed sustained preferential responses to TRβ (HIF2A; Fig. 6C) or to TRα (MYH6 Fig. 6D). Thus, differences in magnitude and kinetics of T_3_ response with the two TRs are reflected at the level of the global T_3_-dependent gene expression program (Figs. 2 and 3) and at individual gene-specific responses (Fig. 6).

### Similar TRα and TRβ Responses in HeLa cells

To extend comparative analysis of TR subtype effects, we examined T_3_ responses in HeLa cells that express exogenous TRs. Here, TR expression was induced by doxycyclin withdrawal for 24 hrs; this regimen elicited optimal TR mRNA induction (Fig. S7). We then treated cells +/− saturating T_3_ (100 nM) for a further 24 hrs.

**Figure 7:**
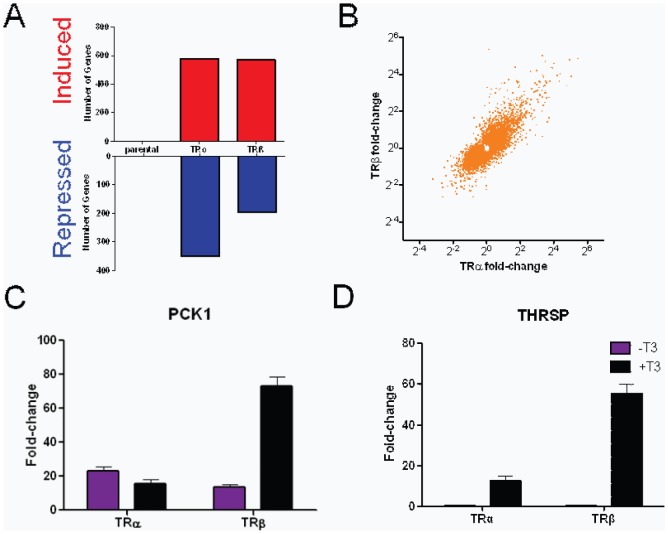
T_3_ Responses in HeLa cells. **A**. Numbers of genes that meet cutoffs for fold T_3_ activation (upper panel, blue) or repression (lower panel, red) in HeLa-TRα and Hela-TRβ cells at 24 hrs treatment, as in Fig. 2. **B**. Plots of fold induction/repression by T_3_ in the presence of TRβ (y-axis) versus TRα (x-axis) in HeLa cells. **C–D**. Representative qPCR analysis showing examples of different gene regulation patterns with the two TRs. **C**, pck1, **D**, thrsp.

More genes responded to T_3_ in the presence of TRs in HeLa cells than HepG2 cells (Fig. 7A). All responses were dependent on exogenous TRs; unlike HepG2 cells, our HeLa cells lack detectable TR protein and transcripts (not shown). Like HepG2 cells, however, the majority of genes were induced by T_3_ and there was near complete overlap between TRα and TRβ target genes; plots of T_3_ responses with TRβ versus TRα again revealed apparent correlation between induction/repression for most genes (Fig. 7B). This extends our conclusion that TRα and TRβ responses are broadly similar and also implies that late TRβ-specific effects observed in HepG2 are a feature of the latter cell type.

Also as seen in HepG2, we detected genes which exhibit preferential responses to TRα or TRβ and verified some effects with qRT-PCR. PCK1 was strongly induced by T_3_ with TRβ but not TRα, although both TR subtypes enhanced transcript abundance without ligand (Fig. 7C). PCK1 was strongly T_3_-dependent in HepG2-TRα and HepG2-TRβ cells (see Fig. 4B) implying that this is a cell-specific effect. More commonly, and similar to HepG2, we observed TR subtype specificity in magnitude of T_3_ response; for example, the *THRSP* gene displayed stronger T_3_ induction with TRβ versus TRα (Fig. 7D).

**Figure 8:**
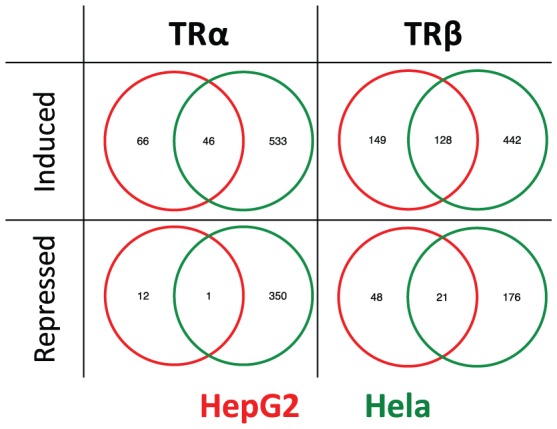
Partial overlap of T_3_ regulated genes in HepG2 and HeLa. Venn diagrams of numbers of T_3_ induced and repressed genes identified in each cell type with TRα and TRβ and overlaps.

Finally, there was only limited overlap between T_3_-regulated gene sets in HepG2 and HeLa (Fig. 8). These differences were not explained by failure to meet cut-offs for fold induction and TRs did regulate different gene sets in the two cell types. This means that strong overlap between TRα and TRβ target genes occurs with two largely distinct gene sets of T_3_ regulated genes in two cell types.

### Unliganded TRα and TRβ Regulate Similar Gene Sets

**Figure 9:**
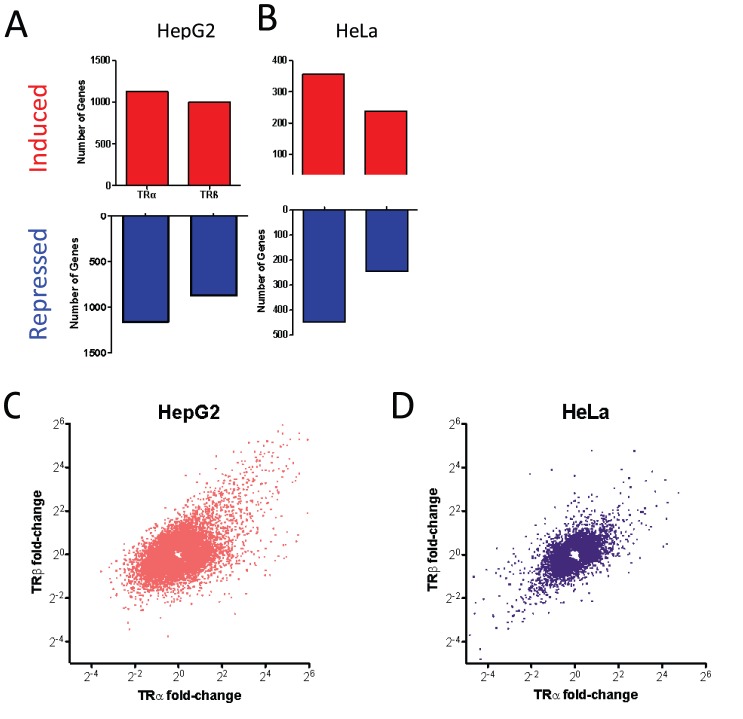
Gene expression changes with unliganded TRs. **A**. Numbers of genes that meet fold cutoffs for activation/repression and statistical significance in response to unliganded TR expression in HepG2 cells, TRα and TRβ expressing cells were compared to parental. **B**. HeLa cells, TRα and TRβ expressing cells after doxycycline withdrawal to induce TRs versus doxycyclin treated cells. Similar results were obtained in comparisons with parental HeLa cells (not shown). **C**. Plots of fold induction/repression by TRβ (y-axis) versus TRα (x-axis) in HepG2 cells. **D**. Plots of fold induction/repression by TRβ (y-axis) versus TRα (x-axis) in HeLa cells.

Since TRs are transcriptionally active without hormone [3], [4], we compared effects of unliganded TRα and TRβ in both cell types (Fig. 9A–D). To do this, we assessed differences in gene expression in HepG2-TR cells versus HepG2 parental cells and parental HeLa cells versus HeLa-TR cells after 24 hrs doxycyclin withdrawal.

Unliganded TRs influenced many genes in both experimental systems (Fig. 9A, B). Expression of more than two thousand genes was altered by the presence of unliganded TRs relative to parental controls in HepG2 cells; with similar numbers up- and down-regulated. Large numbers of genes (≈1000) also responded to short term TR induction in HeLa cells and, again, similar numbers of genes were up and down-regulated. More TR-dependent genes met fold cutoff and statistical significance with unliganded TRα versus TRβ in both cell types. As seen with T_3_ regulation, however, these differences were generally qualitative and not absolute and we observed an essentially linear relationship between induction/repression with unliganded TRα and TRβ in both cell types even though some probe sets suggested preferential response to one of the two TR subtypes (Fig. 9C, D).

### Gene-Specific Variations in Pattern of Response to TRs +/− T_3_

**Figure 10:**
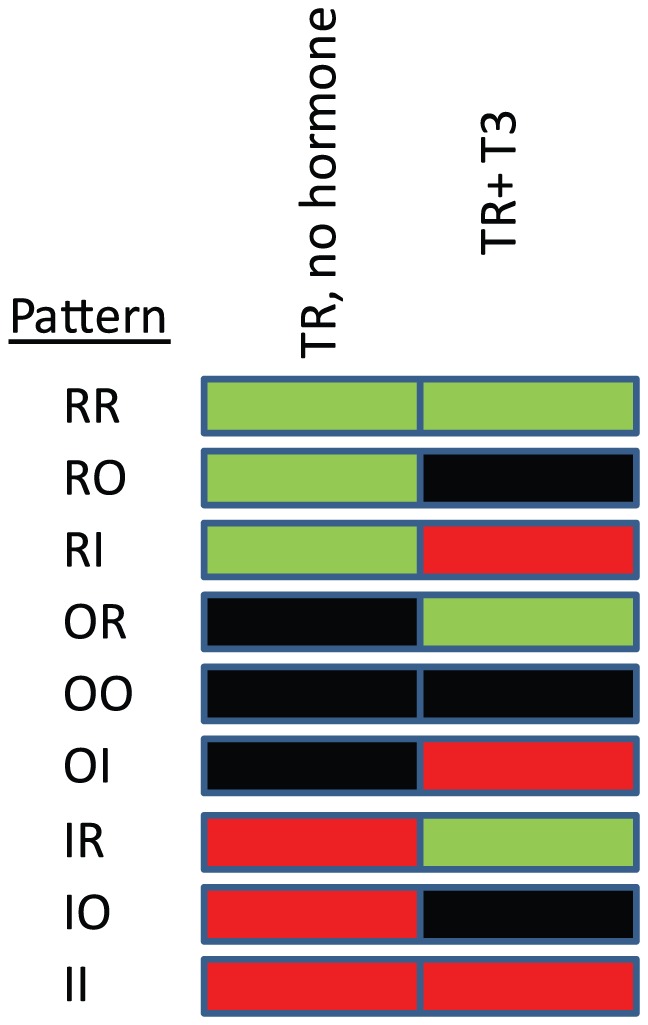
Hypothetical patterns of TR regulation. Genes with statistically significant responses to T_3_ or unliganded TRs were assigned into categories according to net repression (R), induction (I) or no change (O) represented in the schematic heat map. Numbers of genes in each category and overlaps between genes that respond to TRα or TRβ in this manner are shown in Table 1.

Next, we examined specific patterns of target gene regulation by TRs [14]. To do this, we grouped genes with statistically significant responses to unliganded TR or T_3_ into categories according to whether they are repressed (R), unaffected (O) or induced (I) relative to basal gene expression levels in parental cells (Methods and Fig. 10/Table 1/Table S2). In this way, TR and T_3_-dependent genes could be placed into one of eight response patterns shown in the heat map at left. Another category, “OO”, included genes that displayed small changes in response to TR or T_3_ that reached statistical significance, but was assigned to the non-responsive pattern and are not shown (see Methods).

**Table 1:** Patterns of TR regulation.

	HepG2			HeLa		
Pattern	TRα	TRβ	Overlap	TRα	TRβ	Overlap
RR	67	38	7	189	105	37
RO	1587	1737	1071	1129	607	280
RI	202	103	71	462	388	187
OR	124	46	25	1311	1147	598
OI	153	71	49	726	634	303
IR	156	103	44	452	317	124
IO	1011	1305	762	506	323	108
II	36	21	12	112	63	18

Pattern types (see fig. 9) and numbers of genes that conform to each pattern in HepG2 and HeLa with different TRs. Overlaps between genes are shown.

**Figure 11:**
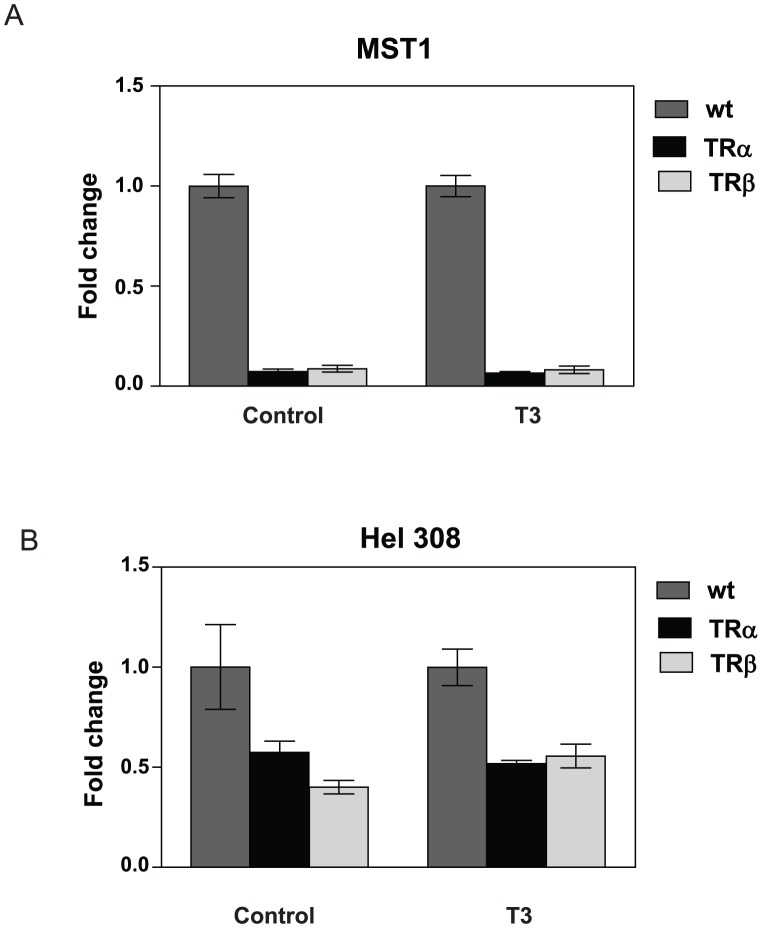
Unusual ligand-independent TR gene-regulation patterns. qPCR verification of genes that display hormone-independent repression by both TRs in HepG2. **A**, mst1, **B**, hel308.

We detected examples of all classes of predicted responses to TR +/− T_3_ (Fig. 10/Table 1/Table S2). As seen in previous study [16], a small percentage of genes were constitutively repressed or induced by TRs (patterns RR and II), and we verified some observations with qRT-PCR (Fig. 11, MST1, hel308 and also see Fig. S4). Other genes were activated or repressed by T_3_, with a majority displaying one of several possible patterns of positive T_3_ response (R0, RI, 0I) and a large minority exhibiting one of several patterns of negative regulation in response to T_3_ (0R,0I, IR). Distributions of genes between different categories of positive and negative response varied with cell type. In HepG2, large majorities of positively and negatively regulated genes grouped into pattern R0 and pattern I0, respectively. Interestingly, these response patterns were mirror images of each other, with unliganded TR repressing positively regulated genes or activating negatively regulated genes and T_3_ reversing these effects. By contrast, the response patterns were more evenly distributed in HeLa.

**Figure 12:**
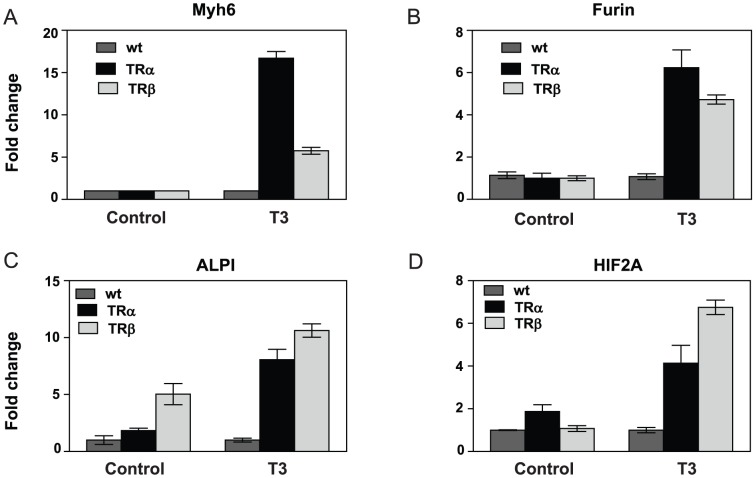
Verification of TR subtype preferences in gene regulation pattern. **A**, Myh6 **B**, furin. Both genes display the same pattern of response to unliganded TRs and T_3_, despite preferential T_3_ induction of Myh6 with TRα. **C**, ALPI, display exclusively ligand-dependent induction with TRα and ligand-dependent induction with TRβ coupled to a strong ligand-independent component. **D**, HIF2A, displays the opposite profile to ALPI in Fig. 12C.

Although TRs regulated similar numbers of genes in the same way, there was only limited overlap between genes that grouped into the same pattern with TRα and TRβ (Fig. 10). This suggests that relatively subtle differences in magnitude of response to TRs +/− T_3_ can translate into different response patterns. We confirmed this impression at a limited set of target genes with qRT-PCR (Fig. 12A–D). For example, Myh6, Furin, ALPI and HIF2A are all induced by T_3_ but Myh6 and furin exhibit the same basic regulation pattern (Fig. 12A, B, pattern 0I), but ALPI is induced by unliganded TRβ (pattern II) and not TRα (pattern 0I) whereas HIF2A is induced by unliganded TRα (Fig. 12C, pattern II)) and not TRβ (Fig. 12D, pattern 0I).

### TR Subtype and Gene Selectivity in T_3_ Dose Response

Finally, we performed a limited survey of relationships between T_3_ dose response, TR subtype and gene (Table 2 and Fig. S8). To do this, we examined effects of varying doses of T_3_ upon selected TR targets in HepG2 cells. Most genes exhibited EC_50_ values in the low nM range (≈1 nM), but TRα exhibited some gene-specific requirements for lower T_3_ concentrations (klf9, pck1) whereas TRβ exhibited gene-specific requirements for higher T_3_ levels (furin, alpi, myh6, hr). The combination of these effects means that many genes exhibit similar T_3_ concentration-dependence in the presence of both TRs whereas others exhibited differential responses to T_3_ with TRα and TRβ, with EC_50_ values varying up to 30-fold when different genes and response to TR subtype are considered. It was noteworthy that higher levels of T_3_ were needed for optimal induction with TRβ in most cases of differential concentration dependence.

**Table 2:** T_3_ concentration dependence of gene induction.

Gene	EC_50_ TRα (nM)	EC_50_ TRβ (nM)
HIF2A	1.661	1.651
HIF1A	3.104	1.158
SLC16A6	0.676	1.408
FURIN	1.004	2.795
ALPI	1.152	4.723
KLF9	0.165	0.846
MYH6	1.220	11.50
PCK1	0.1121	2.037
HR	0.9951	32.20

Table summarizing deduced EC_50_ values for induction of different genes after 24 hrs T_3_ treatment in HepG2-TRα and HepG2-TRβ.

## Discussion

In this study, we compared effects of equal levels of exogenous TRs upon endogenous genes, +/− T_3_, in different cell backgrounds (HepG2 and HeLa) to determine the prevalence of TR subtype specific genes. While hundreds of genes respond to T_3_ or to unliganded TRs in both cell types, and there are differences in degree of response +/− T_3_, we were unable to identify truly TR subtype-specific genes in either cell background; i.e. genes which respond only to one TR subtype and not the other. We conclude that TRα and TRβ regulate the same genes, different from some homologous NR pairs such as the ERs and classic steroid receptors. Our results agree with previous studies of other groups who found that TRs regulate the same genes in mouse liver [14] and in a prior study conducted in HepG2 cells [16].

While TRα and TRβ may regulate the same genes, their actions are far from identical. Part of our analysis overlaps a previous study of similar design by Chan and Privalsky [16], who also stably expressed exogenous TRs in HepG2 cells and found strongly overlapping responses to TRα and TRβ after 6 hrs T_3_ induction but with gene-specific differences in magnitude of response to the two TR subtypes. While our results do not precisely reproduce previous findings, we regard differences as minor and emphasize that there is remarkable concordance between the key points of the two studies. Chan and Privalsky observed fewer genes that responded to T_3_ (≈40 versus 150–300) or unliganded TRs (≈100 versus 1500–2000) and detected a stronger bias towards TRα versus TRβ responses. Both discrepancies are probably explained by differences in TR expression levels. Our comparisons of levels of TR transcripts relative to parental cells in both datasets (not shown) suggest that our cells express more TRs, explaining detection of more TR target genes in our stably transfected HepG2 cells. Moreover, evidence described in the Chan and Privalsky study suggests that their cells may express more TRα than TRβ, explaining observed TRα bias. More importantly, however, both groups conclude that TRs regulate the same genes, all genes flagged as T_3_ regulated in the Chan study appear in our dataset at the 6 hr time point and regulation patterns appear very similar in both analyses (not shown). Our analysis therefore confirms conclusions of this study and, because it also includes extra time points and two cell types, confirms and extends the conclusion that TRs regulate the same genes with differences in precise magnitude of response. Additionally, the fact that we have confirmed that many early responding genes are direct TR targets with CHX treatment indicates that many differential effects must be related to primary differences in direct TR actions.

Our study has also uncovered other ways that TRα and TRβ differ. First, most T_3_ responses tend to be stronger with TRα at earlier times and with TRβ at 24 hrs, although there are numerous counter-examples of individual genes which deviate from this pattern. Second, a large set of late responding highly TRβ-specific genes appears in HepG2 cells. Third, we find that TR subtype preferences in magnitude of T_3_ response may appear at selected time points or persist across several time points. Fourth, differences in magnitude of response +/− T_3_ means that the two TR subtypes exhibit different regulation patterns at individual genes. Finally, we detect variations in T_3_ dose response in a limited survey of target genes and this effect displays a TR subtype-selective component.

Our experiments do not address mechanisms of differential effects, but do permit speculation about possible causes:

1. We suspect that there are fundamental differences in kinetics of T_3_ activation processes in the HepG2 experimental system. We note that: i) faster transcriptional responses to TRα are paralleled by faster T_3_-dependent reductions in steady state TRα levels versus TRβ; this often reflects ubiquitin-dependent turnover of transcriptionally active complexes [31] and ii) some verified early direct TRα targets display similar but slower T_3_ responses with TRβ (see heat map in Figure 5).
2. Early T_3_-dependent changes in gene expression seem to foster an intracellular environment that enhances TRβ actions at some genes; the late HepG2 TRβ-dependent gene set also responds weakly to TRα suggesting that prolonged T_3_ treatment selectively augments TRβ action in these contexts. One possible explanation for this effect is that TRs may induce differential expression or activity of transcription factors that regulate downstream genes and possibly cooperate with TRs in some contexts.
3. We note that TRα and TRβ subtype- and gene-selective actions emerge within the context of wide gene-specific variations in TR action that have also been observed by other groups [14], [16]. We observed that: a) some genes respond to low levels of endogenous TRs in HepG2 parental cells whereas others require exogenous TR expression to mount a detectable response and one gene (ANGPTL4) that is a verified direct TRβ target in parental HepG2 cells [22] is even silenced by TR overexpression, b) magnitude and direction of response to T_3_ and unliganded TRs varies widely, c) there are gene-specific interactions between CHX and T_3_, see Figure 5 d) there are variations in response patterns of both activated and repressed genes +/−T_3_ and this effect displays a cell-specific component and e) T_3_ dose response is gene-specific. We suggest that these gene-specific variations in response reflect gene-context specific variations in mechanisms of TR action and that some of these, in turn, highlight differences in TRα and TRβ function that are not always apparent from standard reporter assays alone. Elucidation of mechanisms of these effects will require better understanding of gene architecture and TR influences upon transcription factor and cofactor recruitment and we propose that systems described within this paper will help us to dissect influences of gene context upon precise mechanisms of TR action.

Finally, it is important to consider whether gene-specific differential actions of TRα and TRβ also occur *in vivo* and possible physiologic impacts of such effects. We know from previously published studies of wild type and TR gene knockout mice that some gene-specific TR behaviors that we have been able to document in culture have direct parallels *in vivo*; these include variations in patterns of TR regulation +/− T_3_ and in T_3_ concentration dependence [14]. This implies that at least some of the gene-specific differential effects seen in our study will also be observed *in vivo*. Indeed, our initial survey of effects of TRβ knockout upon endogenous liver genes in mice revealed that T_3_ response of all genes is diminished but that there are more severe effects at some genes than others (not shown), implying different contributions of TRα and TRβ to T_3_ response.

We can imagine two situations in which gene-specific differential TR actions would be physiologically important. First, TRα and TRβ expression is highly influenced by diurnal rhythm [32]. Thus, variations in TR protein levels could affect the TR target repertoire based upon whether a particular target gene responds preferentially to TRα or TRβ; such differential effects would be observed even without changes in T_3_ levels. Second, TRβ and liver selective thyromimetics (STRMs) such as GC-1 (sobetirome) and KB2115 (eproterome) have been developed to selectively lower serum cholesterol without deleterious effects on heart and combat other aspects of metabolic disease [12]. Some natural TR ligands such as TRIAC are also TRβ selective [23]. Doses of such ligands that activate TRβ but not TRα would be expected to alter gene expression in a TRβ biased manner that would differ from T_3_, which binds the two TRs with similar affinity. It will be interesting to determine whether any such TR subtype and gene-specific effects occur *in vivo* and whether their impact is physiologically or pharmacologically important.

## Supporting Information

Figure S1 TR induction by doxycyclin withdrawal. Western blot showing TRβ expression levels (anti-myc, as in Fig. 1A) after 24 hrs treatment with increasing doxycyclin concentrations up to 20 ng/ml.
(PPTX)

Figure S2 Exogenous TRs are recognized by antibodies against TR primary sequences. We compared TRβ and TRα levels in HepG2-TR cells versus HepG2 cells +/−1 hr T_3_ treatment. The panel shows representative western blots of HepG2-TR and HepG2 cell extracts probed with TR antibodies. Antibodies were TRβ (TRβ72–93; BabCO, Berkeley Antibody Co., now Covance, Richmond, CA, see reference [22]) and TRα (abcam: ab53729). Secondary antibody was goat anti-mouse IgG-HRP conjugate from Santa Cruz Antibodies.
(PPTX)

Figure S3 The TR expression tag does not affect T_3_ response in transient transfection assays. The panel shows luciferase activities at a standard DR-4 reporter with equivalent (optimal) levels of transfected wild type TRβ expression vector or similar flag-TRβ vector.
(PPTX)

Figure S4 Silencing of ANGPTL4 expression by TR overexpression. Results of qPCR analysis to show ANGPTL4 expression levels +/_ T_3_ in parental HepG2 cells versus HepG2-TR cells. Note the silencing of T_3_ response and strong suppression of basal expression levels by unliganded TRs.
(PPTX)

Figure S5 SiRNA directed against the upstream EGFP coding sequences selectively inhibits responses to exogenous TRs. HepG2-TRβ cells were transfected with 5 nM Qiagen negative control siRNA (siRNA_Qiagen-NC) “UUCUCCGAACGUGUCACGU” or and siRNA-EGFP “GCCACAACGUCUAUAUCAUGG”, treated with DMSO (vehicle) or 100 nM T3 24 hr later post siRNA transfection, and RNA was isolated 24 hr later. The left panel shows relative expression of TRβ +/− T3 in the presence of control or EGFP siRNA confirming efficient knockdown of TRβ transcripts in HepG2-TRβ cells (similar knockdown was observed for EGFP). The right panel shows inhibition of T_3_ response at the highly induced C10orf10 gene with the EGFP siRNA treatment and not Qiagen negative control (QNC) siRNA. Similar results were obtained with other T_3_ induced genes in the HepG2-TRβ cells, including anxa1, pck1, slc16a6, and scnn1a. Data presented represents 3 biological replicates, and bars with the same letters are not statistically different (adjusted p-value>0.05; ANOVA, Tukey-HSD on (C10orf10_Cp) - (RPS27A_Cp)).
(PPTX)

Figure S6 Heatmap showing a subset of “indirect” T_3_ target genes, implied by a disruption of the T_3_-response in the presence of CHX. Seventeen probes (genes) of the 158 probes (156 genes) that displayed a≥2-fold fold-change and Benjamini-Hochberg adjusted p-value< = 0.1 (i.e., T_3_ vs. Ctrl treatment) in either HepG2-TRα or HepG2-TRβ cells were determined to be indirect targets based on the compromise of the T_3_-response within the presence of CHX.
(PPTX)

Figure S7 Time course of TR induction in HeLa cells. **A**. Results of qPCR analysis showing optimal induction of TRβ transcripts after 24 hrs DOX withdrawal. **B**. Induced TR is functional as observed in the known TR gene target *thrsp*. Note that expression of this gene is strongly suppressed in response to TR induction within the 24 hr time period of DOX withdrawal.
(PPTX)

Figure S8 Representative concentration dependence curves. qPCR analysis of T_3_ response. 24 hrs treatment with varying amounts of T_3_ shown on the x-axis. Note differences in response curve for KLF9 in the presence of TRα and TRβ.
(PPTX)

Table S1 PCR primer information.
(XLSX)

Table S2 TR regulated gene pattern information.
(XLSX)
